# Association of omphalocele with a patent urachus presenting as a large umbilical cystic mass in a newborn

**DOI:** 10.1002/ccr3.4664

**Published:** 2021-08-23

**Authors:** Roya Farhadi, Seyed Abdollah Mousavi

**Affiliations:** ^1^ Department of Pediatrics Mazandaran University of Medical Sciences Sari Iran; ^2^ Department of Surgery Mazandaran University of Medical Sciences Sari Iran

**Keywords:** omphalocele, patent urachus, umbilical cord, umbilical cyst, urachus

## Abstract

Simultaneous presentation of omphalocele, patent urachus, and umbilical cyst is very rare. There is wide range of differential diagnosis for umbilical cyst. Accurate assessment of umbilical cysts is important to evaluate other abnormalities.

## Graphical abstract

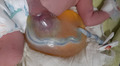

## 1 INTRODUCTION

We present a case of an umbilical mass in a newborn as a rare neonatal anomaly. It is important to know the wide variety of clinical presentation for early detection of associated anomalies and appropriate surgical approach.

A term female neonate, born by Cesarean section, presented with a 10 × 15 cm cystic mass arising from the umbilicus. The mass was translucent and was filled with yellow‐colored fluid. Another 4 × 5 cm mass was visible inside of the large cyst.

**FIGURE 1:**
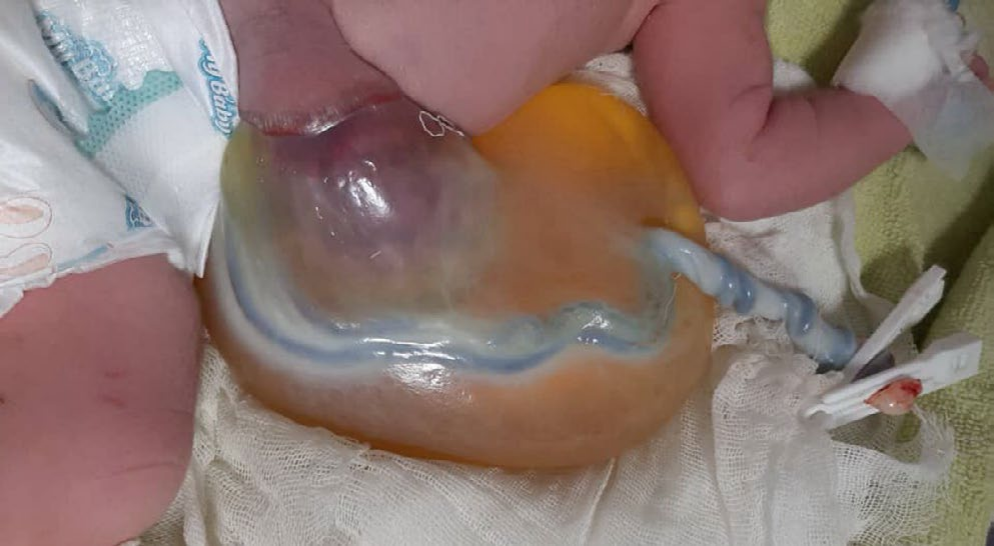
Post‐natal photograph shows the large umbilical cystic mass containing omphalocele

**FIGURE 2:**
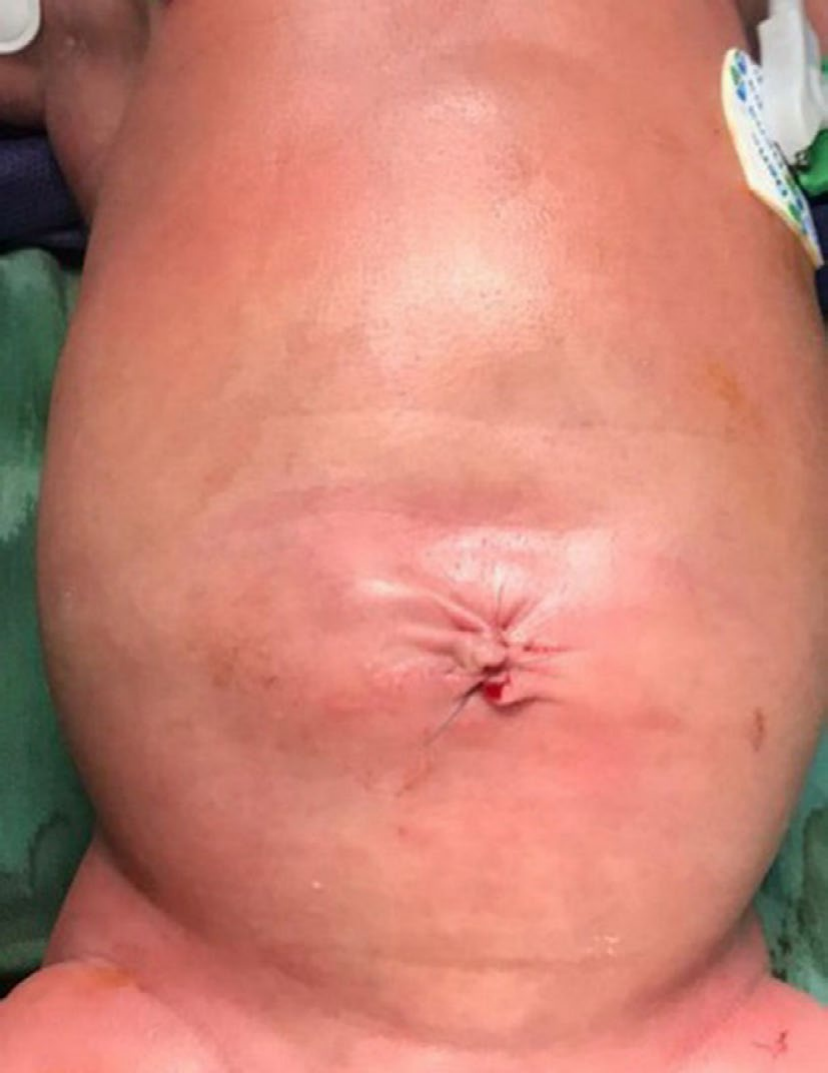
Post‐operative photograph of the newborn: umbilical cyst has been removed, patent urachus was ligated and omphalocele was repaired

The inner mass was determined to be an omphalocele. The baby's intestine protruded through a defect in the abdominal wall near the base of umbilical cord and was covered by a nearly transparent sac. Part of the umbilical cord was along the omphalocele and was located inside the large cyst (Figure 1). Antenatally, the newborn was diagnosed with a giant omphalocele, and karyotype analysis was normal. The baby was referred for surgical repair. She had an omphalocele associated with a patent urachus, which led to leakage of urine into an umbilical cystic mass around omphalocele. The omphalocele was repaired, and the patent urachus was ligated (Figure 2).

Omphalocele concomitant with umbilical cord cysts has infrequently been reported.^1^ Coexistence of cord cyst and patent urachus is very rare as well.^2^ Due to the low incidence of both omphalocele and patent urachus as well as the broad differential diagnosis, it is essential to know the different clinical presentation to guide appropriate management.

## CONFLICT OF INTEREST

None declared.

## AUTHOR CONTRIBUTIONS

RF: wrote and critically reviewed the manuscript and provided the images with description. SAM: was the pediatric surgeon on the case and made final edits.

## ETHICAL APPROVAL

This report for a clinical image was conducted in accordance with the Declaration of Helsinki. Written consent was obtained from the patient's parents.

## Data Availability

Data sharing not applicable to this article as no datasets were generated or analyzed during the current study.
